# Direct interaction between phosphotransferase LpxT and ArnT modulates polymyxin B resistance in *Pseudomonas aeruginosa*

**DOI:** 10.1128/spectrum.01852-25

**Published:** 2026-03-23

**Authors:** Yuxi Bai, Yue Zhao, Yuerong Yuan, Fang Bai, Zhihui Cheng, Weihui Wu, Un-Hwan Ha, Shouguang Jin, Yongxin Jin

**Affiliations:** 1 State Key Laboratory of Medicinal Chemical Biology, Key Laboratory of Molecular Microbiology and Technology of the Ministry of Education, Department of Microbiology, College of Life Sciences, Nankai University 117931, Tianjin, China; 2 Department of Biotechnology and Bioinformatics, Korea University 34973, Sejong, Republic of Korea; Yan'an University, Yan'an, Shaanxi, China

**Keywords:** *P. aeruginosa*, polymyxin B resistance, LpxT, ArnT, PmrB

## Abstract

*Pseudomonas aeruginosa* is a common human opportunistic pathogen that causes a variety of acute and chronic infections. Polymyxins are considered last-resort options for treating serious infections caused by multidrug-resistant gram-negative bacteria. Previously, we evolved PAO1 by serial passage in the presence of sublethal concentrations of polymyxin B, resulting in the polymyxin B-resistant strain PAO1-D14. In this study, we investigate the molecular mechanisms of resistance to polymyxin B in PAO1-D14. We found that the *pmrB*_L189Q_ missense mutation results in a fourfold increase in the minimum inhibitory concentration (MIC), while deletion of *lpxT*, the phosphotransferase-encoding gene, confers an additional eightfold increase in the MIC of the *pmrB*_L189Q_ strain but not in the wild-type PAO1 strain. Using 1-N-phenylnaphthylamine and Dansyl-polymyxin B binding assays, we observed that the *lpxT* mutation decreased the amount of polymyxin B bound to the bacterial surface and reduced membrane permeability in the *pmrB*_L189Q_ mutant. A co-purification assay revealed an interaction between L-Ara4N transferase ArnT and LpxT. The amino acid-substituted version of LpxT_G52AK137A_ lost the interaction with ArnT and no longer restored the polymyxin B resistance of *pmrB*_L189Q_Δ*lpxT*. Additionally, deletion of *arnT* in *pmrB*_L189Q_Δ*lpxT* restored susceptibility to polymyxin B to the levels seen in the *pmrB*_L189Q_ strain. Collectively, our findings reveal a novel role for *lpxT* and its molecular mechanism to defend against polymyxin B in *P. aeruginosa*.

**IMPORTANCE:**

Treatment of *Pseudomonas aeruginosa* infections is challenging due to its antibiotic resistance. Polymyxins are considered last-resort options for treating serious infections caused by multidrug-resistant *P. aeruginosa*. Understanding the molecular mechanisms of polymyxin resistance may provide clues for the development of new therapeutic strategies against *P. aeruginosa*. In this study, we demonstrated that the phosphatidic acid-phosphatase family (PAP2) protein LpxT interacts with L-Ara4N transferase ArnT and controls polymyxin B resistance in *P. aeruginosa*. Our findings reveal a novel role for lpxT and its molecular mechanism to defend against polymyxin B in *P. aeruginosa*.

## INTRODUCTION

*Pseudomonas aeruginosa* is a common human opportunistic pathogen that causes a variety of acute and chronic infections in individuals with impaired immunity, cystic fibrosis, severe burns, and chronic obstructive pulmonary disease (1). Treatment of infections caused by *P. aeruginosa* is challenging due to its high antimicrobial resistance (2). Polymyxin B and polymyxin E (colistin), cationic cyclic peptide antibiotics, represent last-resort options for treating serious infections caused by gram-negative bacteria, including multidrug-resistant *P. aeruginosa* that are resistant to all other available antibiotics (3, 4). However, polymyxin-resistant *P. aeruginosa* has been reported occasionally (4, 5). Therefore, understanding the molecular mechanisms of polymyxin resistance in *P. aeruginosa* is critically important and urgently needed.

Lipopolysaccharide (LPS) constitutes the outermost leaflet of the cell surface bilayer of gram-negative bacteria. As cationic antimicrobial peptides, polymyxins target the negatively charged phosphate groups within the lipid A moiety of LPS at both the outer membrane and the cytoplasmic membrane through ionic interactions, destabilizing the LPS and disrupting cell membrane integrity, ultimately leading to cell death (6–8). In *P. aeruginosa*, the development of polymyxin resistance is genetically attributed to two-component regulatory systems, including PhoP-PhoQ (9), PmrA-PmrB (10), ParR-ParS (11), and CprR-CprS (12). Upon activation, these two-component systems upregulate the *arnBCADTEF* operon, which is responsible for the addition of positively charged 4-amino-4-deoxy-L-arabinose (L-Ara4N) to lipid A, thereby decreasing the negative charge of the bacterial surface and conferring resistance to polymyxins (10–14). It has been reported that aminoarabinosylation of lipid A is critical for the development of polymyxin resistance in *P. aeruginosa* (15, 16). Notably, constitutive activation of these two-component systems can be caused by specific missense mutations in the sensor kinase, leading to bacterial resistance to polymyxins in *P. aeruginosa* (17, 18). The histidine kinase sensor *pmrB* appears to be a commonly mutated gene compared to *pmrA* in polymyxin-resistant *P. aeruginosa* strains (17, 19).

LpxT is a widespread inner membrane enzyme with sequence similarity to members of the phosphatidic acid-phosphatase family (PAP2) (20). LpxT specifically transfers an additional phosphate group to the 1-phosphate group of lipid A within LPS, forming the 1-diphosphate species in some gram-negative bacteria, including *Escherichia coli* and *Salmonella enterica*, thereby increasing the LPS negative charge (20, 21). Unlike LpxT in *E. coli*, LpxT in *P. aeruginosa* adds phosphate groups to both the 1-phosphate and the 4′-position of lipid A (22). Although the role of LpxT in lipid A modification has been demonstrated, its role in polymyxin resistance in *P. aeruginosa* has not yet been elucidated.

In a previous study, we evolved *P. aeruginosa* PAO1 by serial passage in the presence of sublethal concentrations of polymyxin B and obtained a polymyxin B-resistant strain PAO1-D14, which exhibited a 64-fold increase in minimum inhibitory concentration (MIC) when grown in cation-adjusted Mueller-Hinton broth (CA-MHB) medium (19). In this study, we elucidate the molecular mechanisms of resistance to polymyxin B in PAO1-D14. We found that the *pmrB*_L189Q_ missense mutation results in a fourfold increase in MIC, while the *lpxT* mutation confers an additional eightfold increase in the MIC of the *pmrB*_L189Q_ strain but not in the wild-type PAO1 strain. Co-purification assays revealed an interaction between L-Ara4N transferase ArnT and LpxT. The amino acid-substituted version of LpxT_G52AK137A_ lost the interaction with ArnT and no longer restored the polymyxin B resistance of the *pmrB*_L189Q_Δ*lpxT* strain. Additionally, deletion of *arnT* in *pmrB*_L189Q_Δ*lpxT* restored the MIC of polymyxin B to that of the *pmrB*_L189Q_ strain. Our results reveal a novel role for LpxT and its molecular mechanism to defend against polymyxin B in *P. aeruginosa*.

## RESULTS

### Mutation of *lpxT* increases polymyxin B resistance in *pmrB*_L189Q_ mutant

In our previous study, a polymyxin B-resistant strain, PAO1-D14, with a 64-fold increase in MIC was obtained using an experimental evolution assay with sublethal concentrations of polymyxin B (19). Four gene mutations were identified in PAO1-D14 compared to its parental strain, PAO1, with the *pmrB*_L189Q_ mutation conferring a fourfold increase in MIC of polymyxin B (19). The remaining three mutated genes include *ptsP* (*ptsP*_V187G_), which encodes a phosphoenolpyruvate-protein phosphotransferase; *opr86* (*opr86*_Q795R_), which encodes an essential outer membrane (OM) protein, Opr86 (BamA homolog in *E. coli*), that plays a role in OM protein assembly (23); and *lpxT/PA5194* (*lpxT*_Y117fs_), which encodes a PAP2 superfamily kinase that transfers phosphate groups to the lipid A moiety of LPS (22). To test if *lpxT* contributes to the increased resistance to polymyxin B in PAO1-D14, we deleted the *lpxT* gene in the *pmrB*_L189Q_ strain and determined the MIC of polymyxin B using the twofold serial dilution method in both CA-MHB and Lysogeny Broth (LB) medium. As shown in Table 1, the *pmrB*_L189Q_Δ*lpxT* strain exhibited an eightfold increase in MIC of polymyxin B compared to the *pmrB*_L189Q_ strain in both CA-MHB and LB medium. The MIC of polymyxin B in *pmrB*_L189Q_Δ*lpxT* was restored to that of the parental strain, *pmrB*_L189Q_, upon complementation with a wild-type *lpxT* gene. These results suggest that the *lpxT* mutation promotes resistance to polymyxin B in the *pmrB*_L189Q_ strain.

**TABLE 1:** MICs (μg/mL) of polymyxin B for indicated *P. aeruginosa* strain in CA-MHB and LB medium

Strain*^a^*	CA-MHB	LB
PAO1	0.5	0.5
PAO1-D14	32	16
Δ*lpxT*	0.5	0.5
*pmrB*_L189Q_	2	2
*pmrB*_L189Q_Δ*lpxT*	16	16
*pmrB*_L189Q_Δ*lpxT*::*lpxT*	2	2
*pmrB*_L189Q_Δ*lpxT*Δ*arnT*	2	2
Δ*arnT*	0.5	0.5
*pmrB*_L189Q_Δ*arnT*	1	1
*pmrB*_L189Q_Δ*lpxT*Δ*eptA*	16	16
Δ*eptA*	0.5	0.5
*pmrB*_L189Q_Δ*eptA*	2	2
*pmrB*_L189Q_Δ*lpxT*::*lpxT*_G52A_	2	2
*pmrB*_L189Q_Δ*lpxT*::*lpxT*_K137A_	2	2
*pmrB*_L189Q_Δ*lpxT*::*lpxT*_A181G_	2	2
*pmrB*_L189Q_Δ*lpxT*::*lpxT*_G52AK137A_	8	8

^*a*^ PAO1-D14, the evolved polymyxin B-resistance strain; all the mutants were generated in the PAO1 background.

To further examine the role of *lpxT* in resistance to polymyxin B, we generated an *lpxT* deletion mutant in the wild-type PAO1 strain and measured its MIC of polymyxin B. In contrast to the *pmrB*_L189Q_ mutant, deletion of *lpxT* did not affect resistance to polymyxin B in the PAO1 strain (Table 1).

### Mutation of *lpxT* decreases polymyxin B binding and outer membrane permeability in *pmrB*_L189Q_ mutant

**Fig 1:**
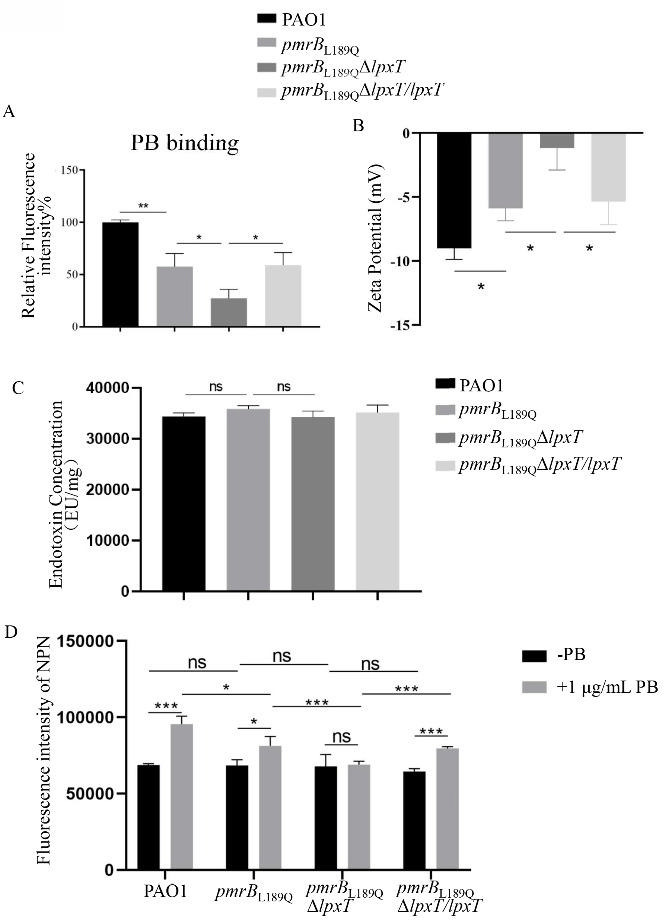
Mutation of *lpxT* decreases polymyxin B binding and outer membrane permeability in the *pmrB*_L189Q_ mutant. (**A**) Dansyl-polymyxin B binding assay. Bacterial cells with an OD_600_ of 1.0 were collected, washed twice with 0.9% NaCl solution, and treated with or without dansyl-polymyxin B for 5 min in the dark. The bacterial cells were washed twice with NaCl solution, and fluorescence intensities were measured with a fluorometer. The data presented are from three independent experiments. *, *P* < 0.05, **, *P* <0.01 (Student’s *t*-test). (**B**) Zeta potential values of indicated strains. *, *P* < 0.05 (Student’s *t*-test). (**C**) Quantitative detection of total LPS extracted from indicated strains by LAL endotoxin assay. ns, not significant (Student’s *t*-test). (**D**) Bacterial cells with an OD_600_ of 1.0 were collected, washed three times with 5 mM GHEPES, and resuspended in the same buffer to an OD_600_ of 0.5. After treatment for 30 min at 25°C with 10 μM 1-N-phenylnaphthylamine (NPN), bacteria were incubated with or without 1 μg/mL polymyxin B for another 30 min at 37°C, and the fluorescence was determined with a fluorometer. ns, not significant; *, *P* < 0.05; ***, *P* < 0.001 (Student’s *t*-test).

The bactericidal activity of polymyxin B is mediated by its initial binding to the lipid A component of LPS (24). To determine whether the increased resistance to polymyxin B in *pmrB*_L189_QΔ*lpxT* was due to decreased interaction between lipid A and polymyxin B, we measured the amount of bacterial surface-associated polymyxin B using dansyl chloride-labeled polymyxin B (25). Previous studies demonstrated that the constitutive activation of *pmrAB*/*pmrB* plays a role in the 4-amino-L-arabinose modification of lipid A, which decreases the negative charge of the cell surface (10, 17). Consistent with this function, the *pmrB*_L189Q_ mutation decreased the amount of polymyxin B associated with the bacterial surface (Fig. 1A). Additionally, the deletion of *lpxT* further decreased the amount of polymyxin B binding to the *pmrB*_L189Q_ strain, while complementation with a wild-type *lpxT* gene restored polymyxin B binding in *pmrB*_L189Q_Δ*lpxT* (Fig. 1A). Furthermore, the zeta potential (ZP) of these strains was determined. As can be seen in Fig. 1B, compared to PAO1, the *pmrB*_L189Q_ mutation exhibited a significant reduction in zeta potential. The deletion of *lpxT* further decreased the zeta potential, which was complemented with a wild-type *lpxT* gene. Next, the total amount of LPS within these strains was determined using the limulus amebocyte lysate (LAL) reagent. As shown in Fig. 1C, the LPS amounts were similar in all the four strains above, suggesting that the decreased binding amount of polymyxin B in *pmrB*_L189Q_Δ*lpxT* was not due to an altered LPS amount.

As cationic antimicrobial peptides, polymyxins destabilize LPS and lead to reduced outer membrane integrity (26). Accordingly, we assessed the OM permeability of wild-type PAO1, *pmrB*_L189Q_, *pmrB*_L189Q_Δ*lpxT*, and the complemented strain *pmrB*_L189Q_Δ*lpxT*::*lpxT* using the hydrophobic fluorescent probe 1-N-phenylnaphthylamine (NPN) (27). All four strains exhibited similar OM permeability (NPN fluorescence levels) in the absence of polymyxin B (Fig. 1D). Upon treatment with 1 μg/mL of polymyxins, OM permeability increased in all strains except for *pmrB*_L189Q_Δ*lpxT*. Importantly, compared to PAO1, OM permeability was significantly decreased in the *pmrB*_L189Q_ strain. Additionally, *pmrB*_L189Q_Δ*lpxT* showed a further decrease in OM permeability compared to *pmrB*_L189Q_ and the complemented strain *pmrB*_L189Q_Δ*lpxT*::*lpxT*, which is consistent with the polymyxin B binding data. These results suggest that the absence of *lpxT* further decreases OM permeability of the *pmrB*_L189Q_ strain under polymyxin B treatment.

### Variation in LpxT contribution to polymyxin B resistance between PAO1 and the *pmrB*_L189Q_ mutant is not due to its altered expression

**Fig 2:**
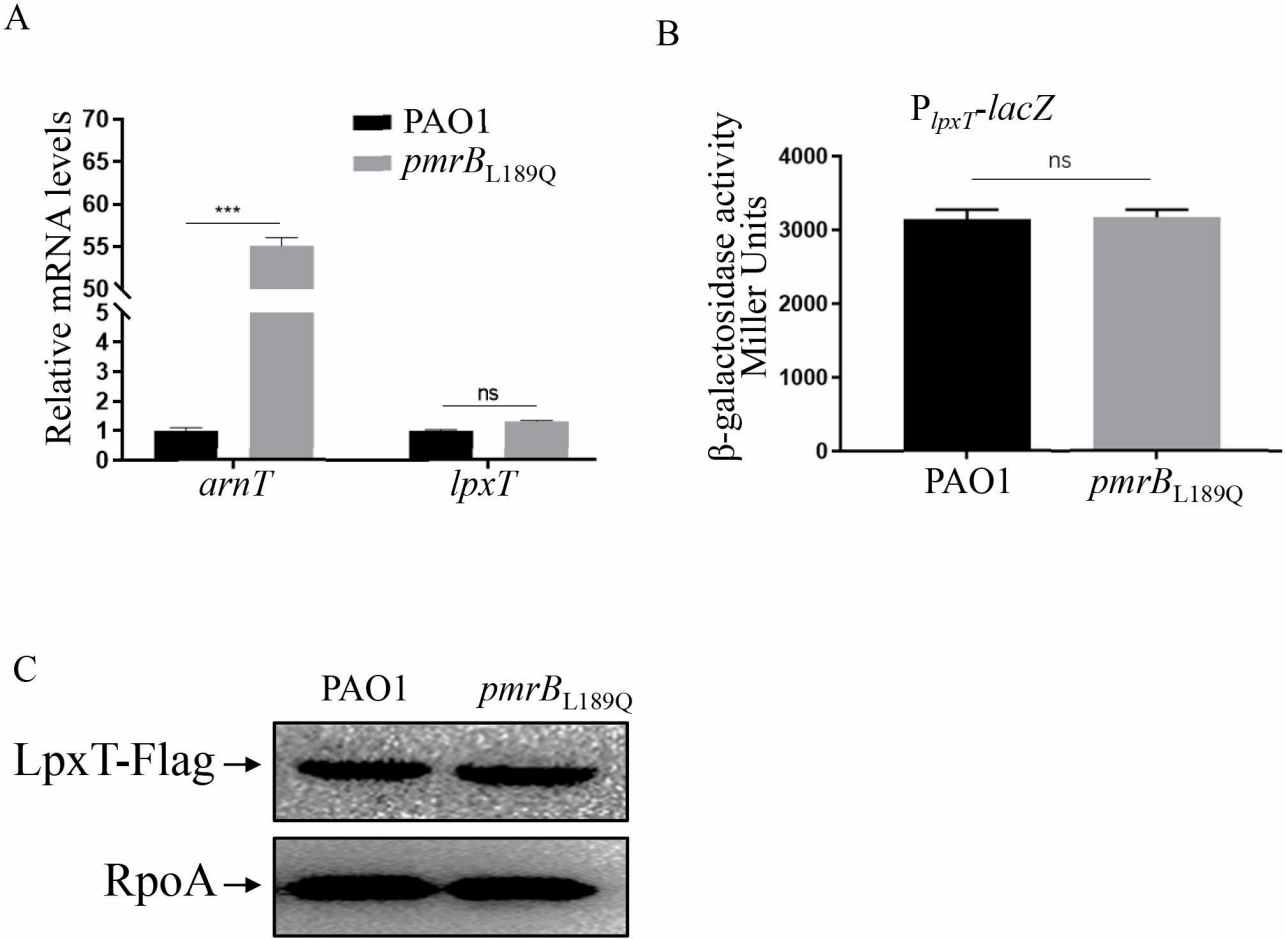
Expression of *lpxT* is similar between PAO1 and the *pmrB*_L189Q_ mutant. (**A**) Relative mRNA levels of *arnT* and *lpxT* in PAO1 and *pmrB*_L189Q_. Total RNA was isolated from bacterial cells with an OD_600_ of 1.0, and the relative mRNA levels of *arnT* and *lpxT* were determined by real-time qPCR using the ribosomal-encoding gene *rpsL* as an internal control. ns, not significant; ***, *P* < 0.001 (Student’s *t*-test). (**B**) PAO1 and *pmrB*_L189Q_ containing the P*_lpxT_-lacZ* transcriptional reporter plasmid were cultured to an OD_600_ of 1.0 in LB and subjected to β-galactosidase assays. Each assay was performed in triplicate, and error bars indicate standard deviations. ns, not significant (Student’s *t*-test). (**C**) Bacterial cells with an OD_600_ of 1.0 were subcultured in LB medium. Protein samples from equivalent bacterial cells were separated on 12% SDS-PAGE gels and probed with Flag or RpoA antibodies.

To understand the mechanism underlying the variation in *lpxT* contribution to polymyxin B resistance between PAO1 and the *pmrB*_L189Q_ mutant, we examined the expression of *lpxT*. The expression of the *arnT* gene has been shown to be controlled by the *pmrAB* two-component system (14). As expected, real-time qPCR results showed a significant upregulation of *arnT* in the *pmrB*_L189Q_ strain (Fig. 2A). However, the relative mRNA level of *lpxT* was unchanged in the *pmrB*_L189Q_ mutant compared to PAO1 (Fig. 2A). To verify the transcriptional level of *lpxT*, a transcriptional fusion of the *lpxT* promoter and a *lacZ* gene (P*_lpxT_-lacZ*) was constructed and introduced into PAO1 and the *pmrB*_L189Q_ mutant. Levels of β-galactosidase were similar between wild-type PAO1 and the *pmrB*_L189Q_ mutant, both in the absence and presence of polymyxin B (Fig. 2B; Fig. S1). To further confirm this observation, we generated a C-terminal Flag-tagged LpxT driven by its native promoter, P*_lpxT_*, with the promoterless pUCP20 plasmid. Consistent with similar transcriptional levels of *lpxT*, similar LpxT-Flag protein levels were detected in wild-type PAO1 and the *pmrB*_L189Q_ mutant (Fig. 2C). These results indicate that the variation in LpxT’s contribution to polymyxin B resistance between PAO1 and the *pmrB*_L189Q_ mutant is not due to altered expression.

### LpxT interacts with ArnT

**Fig 3:**
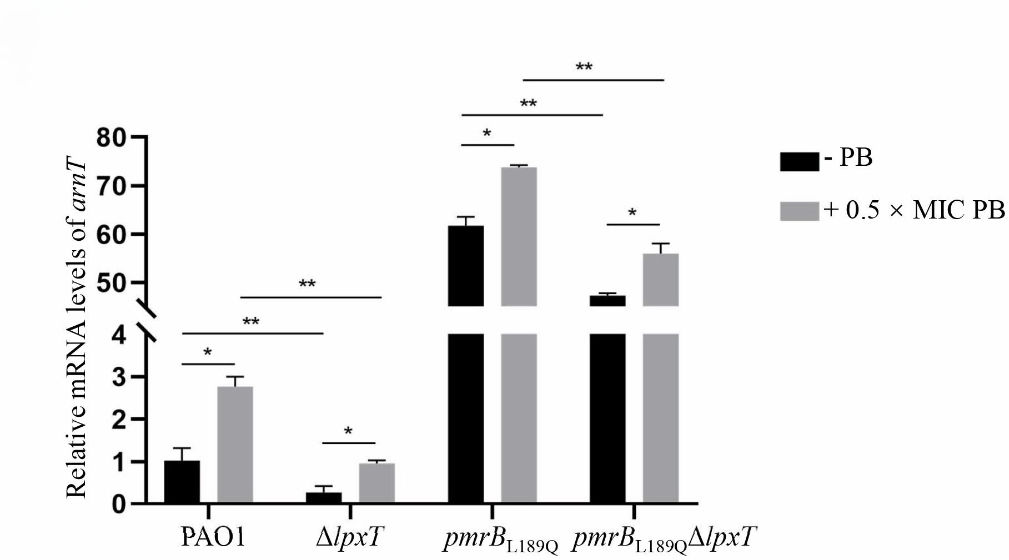
Expression of *arnT* in different strains. Relative mRNA levels of *arnT* in indicated strains with or without polymyxin B treatment. Total RNA was isolated from bacterial cells grown to an OD_600_ of 1.0 with or without 0.5 × MIC polymyxin B, and the relative mRNA levels of *arnT* were determined by real-time qPCR using the ribosomal-encoding gene *rpsL* as an internal control. *, *P* < 0.05; **, *P* < 0.01 (Student’s *t*-test).

Modification of 4-amino-4-deoxy-L-arabinose (L-Ara4N) on lipid A within LPS decreases the electrostatic interaction between the polycationic polymyxins and the phosphate residues of LPS. Since the *pmrB*_L189Q_ mutation increases the expression of *arnT* (Fig. 2A), which encodes L-Ara4N transferase (10), we investigated whether *arnT* expression was further upregulated in *pmrB*_L189Q_Δ*lpxT*. Real-time qPCR results showed that relative mRNA levels of *arnT* were significantly increased when treated with 0.5 × MIC polymyxin B in all four strains, including PAO1, Δ*lpxT*, *pmrB*_L189Q_, and *pmrB*_L189Q_Δ*lpxT* (Fig. 3). As expected, *arnT* mRNA levels were significantly increased in the *pmrB*_L189Q_ mutant compared to the PAO1 strain. However, deletion of *lpxT* did not increase but significantly decreased the relative mRNA levels of *arnT* in both PAO1 and *pmrB*_L189Q_ mutant strains (Fig. 3). These results suggest that *lpxT* does not regulate resistance to polymyxin B in the *pmrB*_L189Q_ mutant through increasing the expression of *arnT*.

**Fig 4:**
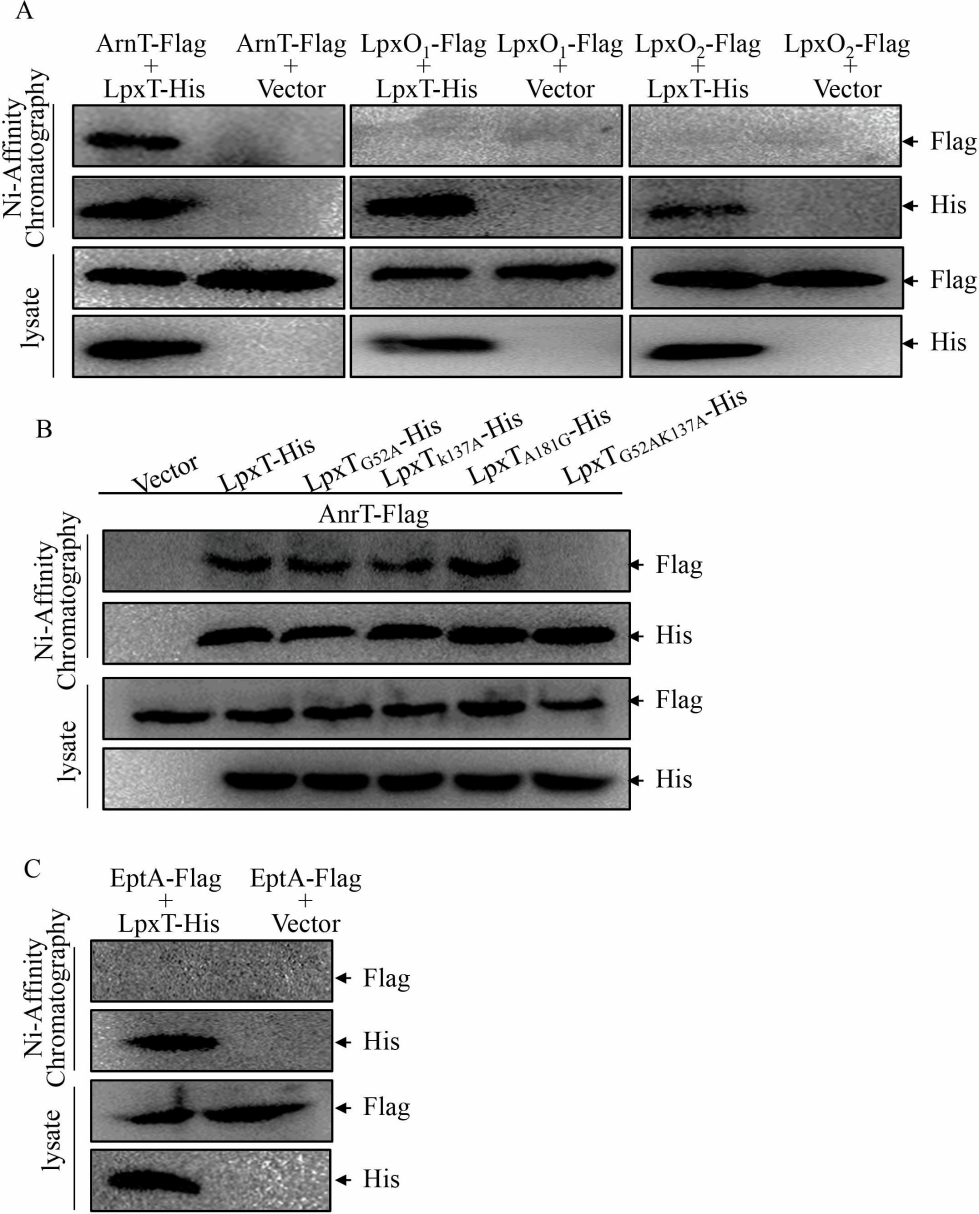
LpxT interacts with ArnT. The PAO1Δ*lpxT* mutant carrying pUCP20-*lpxT*-His with pMMB67EH-*arnT*-Flag, pMMB67EH-*lpxO1*-Flag, or pMMB67EH-*lpxO2*-Flag (**A**), or pMMB67EH-*arnT*-Flag with indicated substituted versions of LpxT (**B**), or pMMB67EH-*eptA*-Flag with LpxT-His or empty vector (**C**) was grown to an OD_600_ of 0.4–0.6 and treated with 1 mM IPTG for 6 h. Bacteria were sonicated and subjected to chromatography with Ni-NTA agarose. His-tagged LpxT and Flag-tagged ArnT, LpxO1, LpxO2, and EptA were detected by Western blot using His and Flag antibodies.

Both ArnT and LpxT are located in the cytoplasmic membrane (www.pseudomonas.com), which provides a possibility for their interaction. To test this possibility, we first predicted the interaction between LpxT and other proteins using the STRING database (https://cn.string-db.org), a pre-computed database for predicting both physical and functional interactions. The top three proteins with the highest confidence scores were lipopolysaccharide biosynthetic protein LpxO2 (0.919 score), ArnT (0.914 score), and lipopolysaccharide biosynthetic protein LpxO1 (0.909 score). Co-purification assays were then performed to determine their interactions. ArnT-Flag, LpxO1-Flag, and LpxO2-Flag fusion proteins were constructed and overexpressed in the Δ*lpxT* mutant carrying an LpxT-His fusion protein. The His-tagged LpxT was purified using Ni-affinity chromatography. As shown in Fig. 4A, ArnT-Flag, but not LpxO1-Flag or LpxO2-Flag, was co-purified with the LpxT-His protein. Another cytoplasmic membrane protein, MexX-His, was utilized as a negative control and could not be co-purified with LpxT-Flag (data not shown). These results demonstrate the interaction between ArnT and LpxT.

To determine whether the interaction between ArnT and LpxT is involved in the LpxT-mediated polymyxin B resistance in *P. aeruginosa*, the critical amino acid sites involved in the interaction were predicted with AlphaFold 3 (https://alphafoldserver.com) and PyMOL. As shown in Fig. S2, Lys137, Gly52, and Ala181 were the critical amino acids of LpxT to interact with ArnT. G52A, K137A, A181G, and G52AK137A substitutions were generated in LpxT. These substitutions had no influence on their expression (data not shown). The interaction between the mutated versions of LpxT and native ArnT was examined with a co-purification assay. As the results shown in Fig. 4B, the LpxT variant with G52A and K137A simultaneous substitutions lost the interaction with ArnT, while the G52A, K137A, or A181G substituted LpxT still had interactions with ArnT. Similarly, the single amino acid-substituted version of LpxT could still complement the MIC of polymyxin B in *pmrB*_L189Q_Δ*lpxT* strain, while the LpxT variant with G52A and K137A simultaneous substitution could not restore the MIC of polymyxin B of *pmrB*_L189Q_Δ*lpxT* to that of the *pmrB*_L189Q_ strain (Table 1).

If the interaction between LpxT and ArnT is involved in the LpxT-mediated regulation of polymyxin B resistance, deletion of *arnT* in *pmrB*_L189Q_Δ*lpxT* should decrease its resistance to polymyxin B. Therefore, we generated a triple mutant, *pmrB*_L189Q_Δ*lpxT*Δ*arnT*, and examined its MIC of polymyxin B. As shown in Table 1, the MIC of polymyxin B in *pmrB*_L189Q_Δ*lpxT*Δ*arnT* was restored to the level of the *pmrB*_L189Q_ mutant, showing an eightfold decrease. However, *arnT* deletion resulted in only a twofold decrease in MIC of polymyxin B in *pmrB*_L189Q_ and had no effect on resistance to polymyxin B in the wild-type PAO1 strain (Table 1).

### EptA is not involved in LpxT-mediated polymyxin B resistance

EptA, responsible for the phosphoethanolamine modification within lipid A, has been reported to compete with LpxT for modification at the 1-phosphate site within LPS in *S. enterica* and *E. coli* (28). Therefore, we further investigated whether EptA is also involved in the LpxT-mediated polymyxin B resistance in *P. aeruginosa*. The co-purification assay showed no interaction between LpxT and EptA (Fig. 4C). In addition, deletion of *eptA* had no effect on the resistance to polymyxin B in the *pmrB*_L189Q_Δ*lpxT* strain (Table 1), indicating that EptA is not involved in LpxT-mediated resistance to polymyxin B in *P. aeruginosa*.

## DISCUSSION

Here, in this study, we identified the molecular basis responsible for the increased resistance to polymyxin B in the PAO1-D14 strain. PAO1-D14 displays a 64-fold increase in the MIC of polymyxin B compared to its ancestral strain PAO1 when grown in CA-MHB medium (19). Four gene mutations were found in PAO1-D14, including *ptsP*_V187G_, *opr86*_Q795R_, *pmrB*_L189Q_, and PA5194_Y117fs_ (*lpxT*_Y117fs_) (19). Simultaneous mutation of *pmrB* and *lpxT* conferred a 32-fold increase in MIC, indicating a possible contribution of *ptsP* or *opr86* to polymyxin B resistance in *P. aeruginosa*. It has been demonstrated that ectopic expression of the *opr86* gene, but not *ptsP*, resulted in a twofold reduction in the MIC of polymyxin B in the evolved polymyxin B-resistant strain (19). Therefore, we postulate that *opr86* might contribute to the remaining resistance to polymyxin B in PAO1-D14. However, the role and molecular mechanism of *opr86* in polymyxin B resistance remain elusive and warrant further study.

The function of *lpxT* has been characterized in *E. coli* and *Salmonella enterica* prior to its study in *P. aeruginosa*. LpxT adds a phosphate group to the 1-phosphate site within lipid A, thereby increasing its negative charge (20, 28). Several positively charged moieties can also be added to the phosphate groups of lipid A, masking the anionic phosphate groups and consequently increasing resistance to cationic antimicrobial peptides. For instance, L-Ara4N and phosphoethanolamine (pEtN) are added to the 4′- and 1- phosphate groups of lipid A in *E. coli* and *S. enterica* by the enzymes ArnT and EptA, respectively (4, 13). It has been demonstrated that EptA competes with LpxT for modification at the 1-phosphate site within LPS in *S. enterica* and *E. coli* (28). PmrR, a small membrane protein, inhibits the phosphotransfer activity of LpxT via direct interaction with LpxT, leaving the lipid A 1-phosphate site open for pEtN modification by EptA (22, 29). In *P. aeruginosa*, which is distinct from *S. enterica* and *E. coli*, the L-Ara4N modification is found to occur at both the 1- and 4′ positions of lipid A phosphate groups (22). Additionally, LpxT can phosphorylate *P. aeruginosa* lipid A at both 1- and 4′-phosphate positions (22). Although the competition between LpxT and ArnT for modification at lipid A phosphate groups has been reported, the detailed molecular basis remains unclear (22). Our study demonstrated that LpxT interacts with ArnT, and this interaction is involved in LpxT-mediated polymyxin B resistance. The interaction between LpxT and ArnT provides a possible explanation for this competition for the same sites of lipid A in *P. aeruginosa* (22). Given that the L-Ara4N and phosphate group addition occurs at the same sites of lipid A in *P. aeruginosa* (22), it is not possible to conclude whether the interaction affects the catalytic activity of ArnT or LpxT, or both, from our present study. Together, this study further suggests that coordinated control occurs in the lipid A modification at the phosphate groups in *P. aeruginosa* (22).

Deletion of the *lpxT* gene alone has no effect on the susceptibility to polymyxin B in the wild-type PAO1 strain, while the absence of *lpxT* results in increased polymyxin B resistance in the *pmrB*_L189Q_ mutant. Our experiments showed that the expression of *lpxT* was similar in the *pmrB*_L189Q_ mutant compared to PAO1, consistent with a previous study reporting that transcription of *lpxT* was not induced under *pmrAB*-inducing conditions (low Mg^2+^) (22). Therefore, the differential contribution of *lpxT* to polymyxin B resistance is not due to its altered expression between PAO1 and *pmrB*_L189Q_. Given that *arnT* is upregulated in the *pmrB*_L189Q_ mutant and that ArnT interacts with LpxT, as well as that *arnT* deletion restores the MIC of polymyxin B in *pmrB*_L189Q_Δ*lpxT* to that in *pmrB*_L189Q_, we propose that *lpxT* regulates polymyxin B resistance in *pmrB*_L189Q_ through interaction with ArnT. In the wild-type PAO1, the expression level of *arnT* is low; therefore, deletion of *arnT* or its interacting protein gene *lpxT* does not affect the polymyxin B resistance (Table 1). In contrast, in *pmrB*_L189Q_, where *arnT* expression is high and LpxT’s binding blocks partially ArnT, deletion of *lpxT* increases polymyxin B resistance. Deletion of *arnT* further reverses the increased resistance in *pmrB*_L189Q_Δ*lpxT*.

Interestingly, expression of *arnT* was downregulated in the absence of *lpxT* in both wild-type PAO1 and *pmrB*_L189Q_ mutant. Transcription of *arnT* is primarily activated by the two-component systems, including PmrAB in *P. aeruginosa* (10). It is possible that the absence of LpxT alters the modification of Lipid A and membrane stability, subsequently influencing *arnT* expression through the two-component systems. Further investigation is needed to elucidate the detailed molecular mechanisms.

Although this is the first report on the molecular mechanism of polymyxin B resistance mediated by LpxT in *P. aeruginosa*, *lpxT* mutation has been found to associate with increased colistin resistance when combined with other mutations in the experimental evolution of *P. aeruginosa* lab strains PA14 or PAO1 (16, 30). Of note, a missense mutation in *lpxT* has also been found in the resistant mutant to murepavadin, a peptidomimetic antibiotic targeting LPS transport protein D (LptD) (31). Therefore, it is worthwhile to investigate clinical *P. aeruginosa* isolates with high-level polymyxin resistance for possible mutations in the *lpxT* gene in addition to *pmrAB*.

Interestingly, deletion of both *arnT* and *lpxT* in the *pmrB*_L189Q_ mutant restores polymyxin B susceptibility to the level of the *pmrB*_L189Q_ mutant, but not of the wild-type PAO1. And also, deletion of *arnT* in the *pmrB* mutant did not restore polymyxin B susceptibility to wild-type levels, but conferred a twofold MIC of wild-type PAO1. We postulated that other factors might be involved in the PmrAB-mediated polymyxin resistance in addition to Ara4N modification within Lipid A in *P. aeruginosa*. Of note, it had been reported that PmrAB activation resulted in the modification of phosphate groups of the lipid A with ethanolamine, and of the 4′ phosphate of lipid A with aminoarabinose in *S. typhimurium* (32).

## MATERIALS AND METHODS

### Bacterial strains and plasmids

The bacterial strains, plasmids, and primers used in this study are listed in Tables S1 and S2. Bacterial cells were grown at 37°C in Lysogeny Broth (LB) medium (5 g/L NaCl, 5 g/L yeast extract, and 10 g/L tryptone) with shaking at 200 rpm or on LB agar plates (LB medium containing 15 g/L agar) at 37°C, unless indicated. To maintain plasmids, antibiotics were added to the medium at the following concentrations: for *P. aeruginosa*, tetracycline at 50 μg/mL, gentamicin at 50 μg/mL, and carbenicillin at 150 μg/mL; for *E. coli*, tetracycline at 10 μg/mL, gentamicin at 10 µg/mL, and ampicillin at 100 μg/mL. When needed, IPTG (isopropyl β-D-1-thiogalactopyranoside) was added to the medium at a final concentration of 1 mM.

### Construction of plasmids and bacterial strains

For gene complementation, the specific gene was amplified by PCR using specific primers (Table S2) with PAO1 genomic DNA as the template. The resulting PCR product was digested with restriction endonucleases and inserted into the corresponding vector to generate the recombinant plasmid. Gene deletions or point mutations in *P. aeruginosa* were generated by homologous recombination using the suicide plasmid pEX18Tc (33). DNA fragments corresponding to the upstream and downstream regions of the target gene were individually amplified by PCR using specific primer pairs UF/UR and DF/DR (Table S2), digested with appropriate restriction endonucleases, and directionally cloned into pEX18Tc, resulting in the deletion construct. The deletion construct was transferred into *P. aeruginosa* via conjugation with *E. coli* S17, and gene deletion was conducted using a *sacB*-based strategy as previously described (33). The target deletion mutant was verified by PCR and sequencing analysis.

To generate strain *pmrB*_L189Q_∆*lpxT*::*lpxT*_G52A_, the G52A point mutation was introduced into the reverse complementary primers *lpxT*_G52A_-F and *lpxT*_G52A_-R. Two DNA fragments were PCR amplified with primer pairs *lpxT*_mut_-F/*lpxT*_G52A_-R and *lpxT*_mut_-R/*lpxT*_G52A_-F, and cloned into pUC18T-miniTN7T utilizing homologous recombination-based gene cloning methods. Then the resultant plasmid was introduced into the chromosome of the *pmrB*_L189Q_∆*lpxT* strain by electroporation along with the helper plasmid pTNS3. *pmrB*_L189Q_∆*lpxT*::*lpxT*_K137A_, *pmrB*_L189Q_∆*lpxT*::*lpxT*_A181G_, and *pmrB*_L189Q_∆*lpxT*::*lpxT*_G52AK137A_ were constructed with similar manipulations. For pUCP20-*lpxT*_G52A_-His, the two DNA fragments, amplified with the same primers pairs *lpxT*_mut_-F/*lpxT*_G52A_-R and *lpxT*_mut_-R/*lpxT*_G52A_-F, were used as template for amplification of *lpxT*_G52A_ DNA fragment. The resultant DNA fragments were digested with *Eco*RI-*Hin*dIII and then cloned into pUCP20. pUCP20-*lpxT*_K137A_, pUCP20-*lpxT*_A181G_, and pUCP20-*lpxT*_G52AK137A_ were generated with a similar strategy.

### Determination of the MIC against polymyxins

The MICs of polymyxin B were determined by the twofold serial dilution method using cation-adjusted Mueller-Hinton broth (CA-MHB) as previously described (34). Overnight bacterial cultures were 50-fold diluted into fresh LB medium and grown to an OD_600_ of 1.0. Polymyxins were serially diluted in CA-MHB (100 μL) in a 96-well plate. Then, 100 μL of CA-MHB containing 10^6^ CFU/mL cells were added to each well containing the serially diluted antibiotics. The plates were incubated at 37°C for 18–24 h. The MICs were determined as the lowest antibiotic concentration with no visible bacterial growth.

### Assessment of cell membrane permeability

The integrity of the cell membrane was assessed by the fluorescent probe 1-N-phenylnaphthylamine (NPN) with minor modifications (27). Briefly, overnight cultures of bacterial cells were diluted 50-fold into fresh LB medium and cultured to an OD_600_ of about 1.0 at 37°C. The bacterial cells were washed three times with GHEPES buffer (5 mM HEPES containing 5 mM glucose, Caisson Labs). The bacterial cells were then resuspended and standardized to an OD_600_ of 0.5 in the GHEPES buffer. NPN probe (Macklin) was added to the cells at a final concentration of 10 μM and incubated for 30 min at 25°C. Then, 0 or 1 μg/mL polymyxin B was added to the bacterial suspension and incubated for another 30 min at 37°C. The fluorescence was determined at excitation/emission wavelengths of 350/420 nm using a fluorometer (Varioskan Flash; Thermo Scientific). All tests were carried out in triplicate.

### Dansyl-polymyxin B binding assay

Dansyl-labeled polymyxin B was synthesized and used for the polymyxin B binding assay as described previously (9, 35). Cells of the indicated bacteria, at an OD_600_ of about 1.0, were collected by centrifugation, washed twice with 0.9% NaCl solution, and standardized to 10^9^ CFU/mL in the same solution. The bacteria were then incubated with or without dansyl-polymyxin B at room temperature for 5 min in the dark. The bacteria were then washed twice and resuspended in 1 mL 0.9% NaCl solution. A total of 150 μL of each suspension was added to each well of a black 96-well microtiter plate (Nunc). The fluorescence was measured at excitation/emission wavelengths of 340/485 nm using a fluorometer (Varioskan Flash; Thermo Scientific). The relative fluorescence intensity of the cell-bound dansyl-polymyxin B was calculated as [100 × (F − F_0_) / (F − F_0_) of PAO1] %. F_0_ represents the fluorescence intensity of samples without dansyl-polymyxin B; and F represents the fluorescence intensity of bacteria with dansyl-polymyxin B.

### Measurement of zeta potential (ZP)

Zeta potential (ZP) was determined as described previously (36, 37). Bacterial cells, at an OD_600_ of about 1.0, were collected, washed twice with sterile 0.9% NaCl solution (pH 7.0), and standardized to 5 × 10^8^ CFU/mL in the same solution. The cell suspensions were further diluted 1,000-fold, and ZP was determined at 25°C using a Zetasizer Nano-ZS90 instrument (Malvern Instruments Inc, Westborough, MA, USA). The technique used for the ZP measurements was in accordance with the Malvern laser Doppler velocimetry patterns coupled with M3-phase analysis light scattering. The experiment was conducted in triplicate for each sample.

### Quantitative measurement of LPS

Overnight bacterial cultures were diluted at 1:50 into fresh LB medium and grown to an OD_600_ of 1.0 at 37°C. Total LPS was extracted with lipopolysaccharide extraction kit (Solarbio, EX1740) following the manufacturer’s instructions, and then subjected to LPS quantitation using a Toxinsensor Chromogenic LAL Endotoxin Assay Kit (GenScript, cat. no.: L00350C) following the kit’s instructions.

### Western blot analysis

A single colony of the indicated bacterial strain was inoculated into LB medium containing carbenicillin, grown overnight, diluted 50-fold into fresh LB medium, and then cultivated to an OD_600_ of about 1.0 with shaking at 200 rpm. Samples from an equivalent number of bacterial cells were collected, mixed with loading buffer, incubated at 99°C for 10 min, and then loaded onto and separated by a 12% sodium dodecyl sulfate-polyacrylamide (SDS-PAGE) gel. Proteins were then transferred onto a polyvinylidene difluoride membrane (Millipore), probed with a primary antibody against Flag or RpoA (BioLegend) for 1 h at room temperature, and then incubated with a secondary antibody for 1 h at room temperature. Signals were detected using an ECL Plus kit (Millipore) and visualized using a Bio-Rad molecular imager (ChemiDoc XRS).

### RNA isolation and real-time qPCR

Overnight bacterial cultures were diluted 1:50 into 3 mL fresh LB medium with or without 0.5 × MIC polymyxin B, and cultivated to an OD_600_ of about 1.0. Total RNA was isolated using the bacterial total RNA kit (Zomanbio) according to the manufacturer’s instructions. The RNA concentrations were determined using a NanoDrop spectrophotometer. One microgram of purified RNA was used to synthesize cDNA with random primers and PrimeScript Reverse Transcriptase (Vazyme). The resulting cDNA was diluted by adding 100 μL ddH_2_O. cDNAs were mixed with specific qPCR primers (Table S2), SYBR Premix Ex (Vazyme), and ddH_2_O for real-time qPCR. Reactions were conducted in a CFX Connect real-time PCR machine using the manufacturer-provided software. *rpsL*, the 30S ribosomal protein-coding gene, was utilized as an internal control.

### β-Galactosidase assay

A single colony of bacteria was inoculated in LB medium and grown overnight at 37°C. Bacterial cultures were diluted 50-fold into fresh medium with or without polymyxin B and cultivated to an OD_600_ of about 1.0. One and a half milliliters of the bacterial culture was collected and resuspended in 1.5 mL of Z buffer (0.06 M Na_2_HPO_4_, 0.04 M NaH_2_PO_4_, 0.01 M KCl, 0.001 M MgSO_4_, and 0.05 M β-mercaptoethanol). A 1 mL aliquot of the suspension was used to measure OD_600_. The remaining 500 μL of the suspension was mixed with 10 μL of chloroform and 10 μL of 0.1% sodium dodecyl sulfate (SDS), followed by vortexing for 10 s. Then, 100 μL of ortho-nitrophenyl-β-galactopyranoside (4 mg/mL) was added to the reaction and incubated at 37°C. When the color of the mixture turned light yellow, 500 μL of Na_2_CO_3_ (1 M) was added to stop the reaction, followed by centrifugation and measurement of OD_420_. The β-galactosidase activities were calculated as: Miller units = (1,000 × OD_420_) / (T × V × OD_600_) [T represents reaction time (minutes); V presents volume of bacterial culture].

### Co-purification assay

∆*lpxT* bacterial strains containing the indicated plasmid pairs were grown overnight, diluted 50-fold into fresh LB medium, and then further grown to an OD_600_ of 0.4–0.6. IPTG was added to the medium at a final concentration of 1 mM to induce expression of proteins in plasmid pMMB67EH for 6 h. Bacterial cells (OD_600_ about 2.0) were collected, resuspended in 1 mL lysis buffer (46.6 mM Na_2_HPO_4_, 3.4 mM NaH_2_PO_4_, 0.3 M NaCl, pH 8.0), and lysed by sonication. The lysates were collected by centrifugation and incubated with 50 μL Ni-Affinity Chromatography beads for 1 h at 4°C. The Ni-Affinity Chromatography was then collected and washed six times with 1 mL washing buffer (lysis buffer containing 20 mM imidazole). Samples were eluted with 80 μL elution buffer (lysis buffer with 300 mM imidazole) and mixed with 20 μL loading buffer. Co-purified proteins in the samples were examined by Western blot assay.

### Statistical analysis

GraphPad Prism 9.0 software was used for statistical analyses. *P*-values were calculated using the two-tailed unpaired Student’s *t*-test. Differences were considered statistically significant when the *P*-value was below 0.05.

## SUPPLEMENTAL MATERIAL

Supplemental material spectrum.01852-25-s0001.pdf Fig. S1 and S2; Tables S1 and S2.

ASM does not own the copyrights to Supplemental Material that may be linked to, or accessed through, an article. The authors have granted ASM a non-exclusive, world-wide license to publish the Supplemental Material files. Please contact the corresponding author directly for reuse.
