# Supplementary material for: Direct interaction between phosphotransferase LpxT and ArnT modulates polymyxin B resistance in Pseudomonas aeruginosa
Source: Microbiol Spectr. 2026 Mar 23;14(5):e01852-25. doi: 10.1128/spectrum.01852-25 (PMC13142001; doi:10.1128/spectrum.01852-25)
Supplement: Supplemental material — Fig. S1 and S2; Tables S1 and S2. [file spectrum.01852-25-s0001.pdf]

Fig. S1

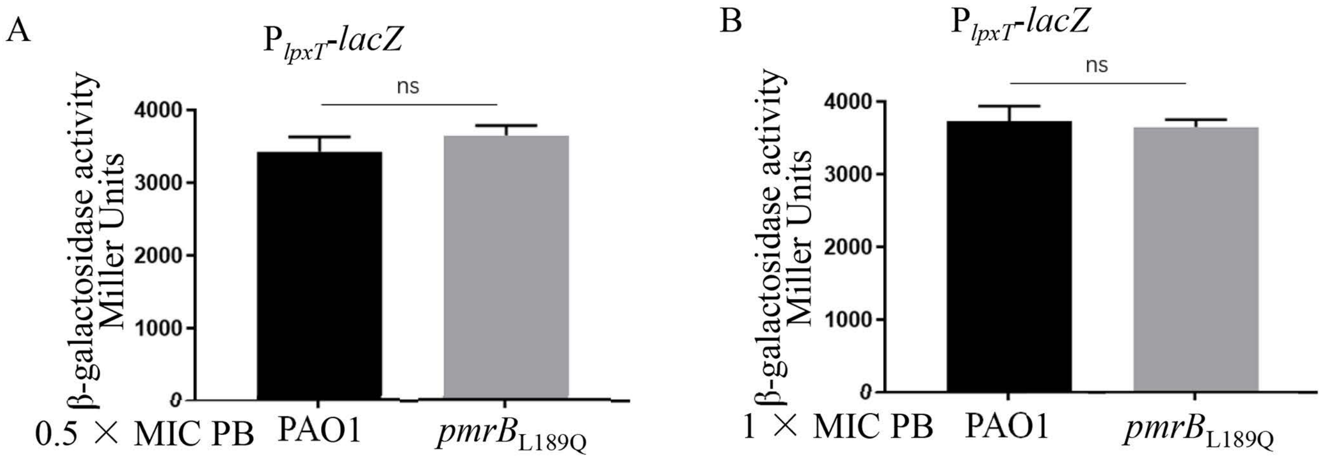

Fig. S1. Transcription of *lpxT* in the presence of polymyxin B. PAO1 and *pmrB*$_{L189Q}$ containing the P$_{lpxT}$-*lacZ* transcriptional reporter plasmid were cultured to an OD_{600} of 1.0 in LB with indicated concentration of polymyxin B, and subjected to β-galactosidase assays. Each assay was performed in triplicate, and error bars indicate standard deviations. ns, not significant; by Student's *t*-test.

Fig. S2

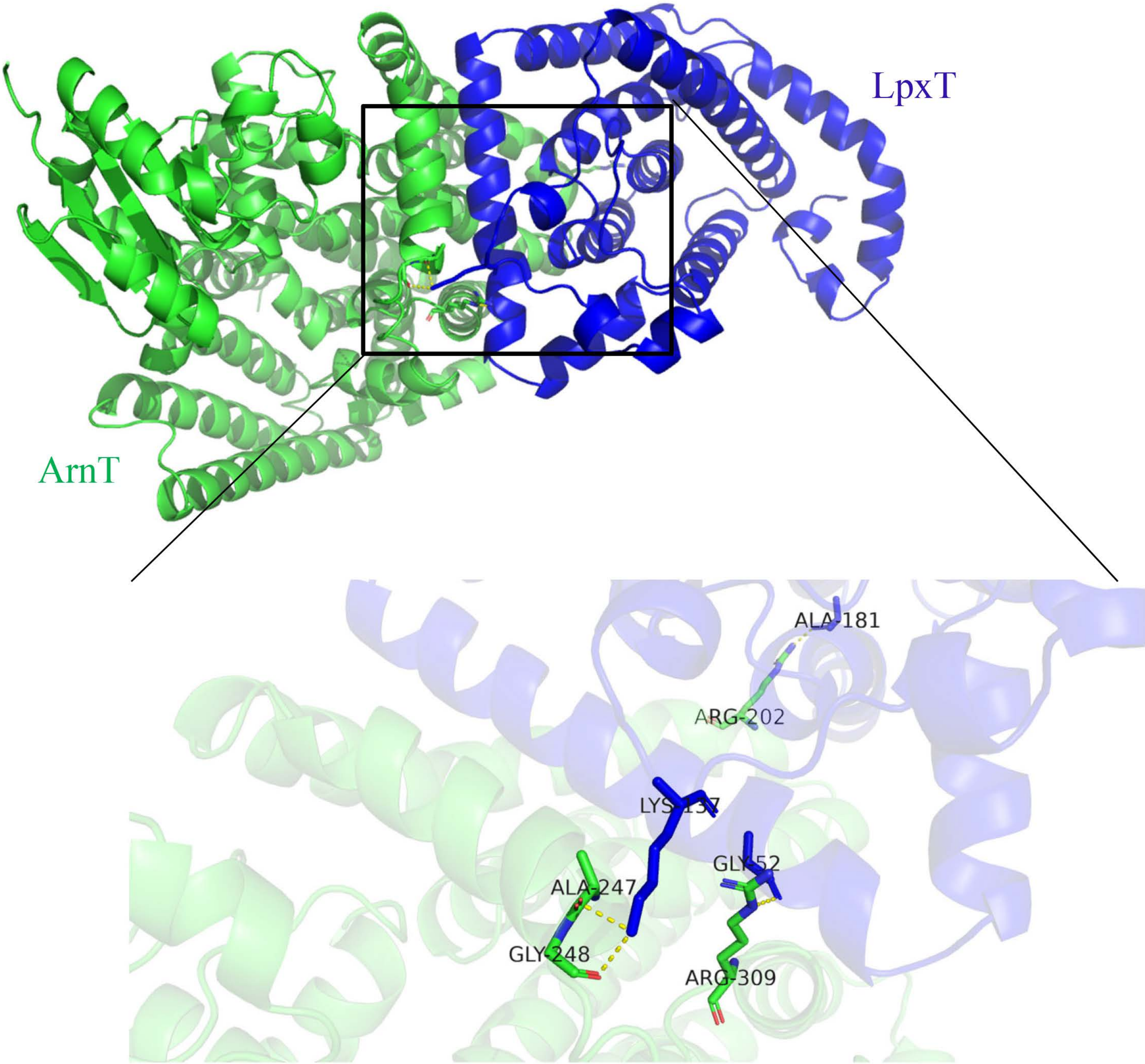

Fig. S2. The interaction between LpxT and ArnT predicted with PyMOL and AlphaFold 3 (https://alphafoldserver.com/).

Table S1. Bacterial strains and plasmids used in this study.

Strains or plasmids	Description	Source or reference
**strains**		
DH5α	F$^-$ ϕ 80d*lacZ*ΔM15 *endA1 recA1 hsdR17*(r$_K^-$ m$_K^+$) *supE44 thi-1 relA1* Δ(*lacZYA-argF*)*U169 gyrA96 deoR*	TransGen
S17-1	RP4-2 Tc::Mu Km::Tn*7* Tp^r Sm^r Pro Res$^-$ Mod$^+$	Stratagene
PAO1	Wild type	[1]
PAO1-D14	Serial passage of PAO1 for 14 days under the sub-MIC of polymyxin B	[1]
pmrB$_{L189Q}$	*pmrB*$_{L189Q}$ variant of PAO1	This study
pmrB$_{L189Q}$Δ*lpxT*	*lpxT* deletion mutant of *pmrB*$_{L189Q}$ strain	This study
pmrB$_{L189Q}$Δ*lpxT*::*lpxT*	Experession *lpxT* with pUC18T-miniTn7T plasmid in *pmrB*$_{L189Q}$Δ*lpxT* strain	This study
pmrB$_{L189Q}$Δ*lpxT*::*lpxT*$_{G52A}$	Experession *lpxT*$_{G52A}$ with pUC18T-miniTn7T plasmid in *pmrB*$_{L189Q}$Δ*lpxT* strain	This study
pmrB$_{L189Q}$Δ*lpxT*::*lpxT*$_{K137A}$	Experession *lpxT*$_{K137A}$ with pUC18T-miniTn7T plasmid in *pmrB*$_{L189Q}$Δ*lpxT* strain	This study
pmrB$_{L189Q}$Δ*lpxT*::*lpxT*$_{A181G}$	Experession *lpxT*$_{A181G}$ with pUC18T-miniTn7T plasmid in *pmrB*$_{L189Q}$Δ*lpxT* strain	This study
pmrB$_{L189Q}$Δ*lpxT*::*lpxT*$_{G52AK137A}$	Experession *lpxT*$_{G52AK137A}$ with pUC18T-miniTn7T plasmid in *pmrB*$_{L189Q}$Δ*lpxT* strain	This study
Δ*lpxT*	*lpxT* deletion mutant of PAO1	This study
Δ*arnT*	*arnT* deletion mutant of PAO1	This study
pmrB$_{L189Q}$Δ*arnT*	*arnT* deletion mutant of *pmrB*$_{L189Q}$ strain	This study
pmrB$_{L189Q}$Δ*lpxT*Δ*arnT*	*lpxT-arnT* double mutant of *pmrB*$_{L189Q}$ strain	This study
Δ*eptA*	*eptA* deletion mutant of PAO1	This study
pmrB$_{L189Q}$Δ*eptA*	*eptA* deletion mutant of *pmrB*$_{L189Q}$ strain	This study
pmrB$_{L189Q}$Δ*lpxT*Δ*eptA*	*lpxT-eptA* double mutant of *pmrB*$_{L189Q}$ strain	This study
**Plasmids**		
pUCP20	shuttle vector between *E. coli* and *P. aeruginosa*; Amp^r	[2]
E1553-*lpxT*-Flag	Flag-tagged *lpxT* gene with its native promoter from PAO1 in promoterless pUCP20; Amp^r	This study
pUCP20-*lpxT*-His	*lpxT* gene from PAO1 with His tag in pUCP20; Amp^r	This study
pMMB67EH	shuttle vector pMMB67EH between *E. coli* and *P. aeruginosa*; Gm^r	[3]
pMMB67EH-*arnT*-Flag	*arnT* gene from PAO1 with Flag tag in pMMB67EH; Gm^r	This study
pMMB67EH-*lpxO1*-Flag	*lpxO1* gene from PAO1 with Flag tag in	This study

	pMMB67EH; Gm^r	
pMMB67EH-*lpxO2*-Flag	*lpxO2* gene from PAO1 with Flag tag in pMMB67EH; Gm^r	This study
pMMB67EH-*eptA*-Flag	*eptA* gene from PAO1 with Flag tag in pMMB67EH; Gm^r	This study
pUCP20-$lpxT_{G52A}$	*lpxT* gene with G52A mutant in pUCP20, Amp^r	This study
pUCP20-$lpxT_{K137A}$	*lpxT* gene with K137A mutant in pUCP20, Amp^r	This study
pUCP20-$lpxT_{A181G}$	*lpxT* gene with A181G mutant in pUCP20, Amp^r	This study
pUCP20-$lpxT_{G52AK137A}$	*lpxT* gene with G52A and K137A double mutant in pUCP20, Amp^r	This study
pEX18Tc	gene knockout vector; Tc^r	[4]
pEX18Tc-*lpxT*	*lpxT* gene deletion on pEX18Tc; Tc^r	This study
pEX18Tc-$pmrB_{L189Q}$	$pmrB_{L189Q}$ mutant gene on pEX18Tc; Tc^r	This study
pEX18Tc-*arnT*	*arnT* gene deletion on pEX18Tc; Tc^r	This study
pEX18Tc-*eptA*	*eptA* gene deletion on pEX18Tc; Tc^r	This study
P_{lpxT}-*lacZ*	pDN19*lacZ*Ω with *lpxT* promoter; Tc^r	This study
pUC18T-miniTn7T-*lpxT*	*lpxT* gene from PAO1 in pUC18T-miniTn7T; Gm^r	This study

1. Yang, F., et al., *MvfR Controls Tolerance to Polymyxin B by Regulating rfaD in Pseudomonas aeruginosa.* Microbiol Spectr, 2023. **11**(3): p. e0042623.
2. West, S.E., et al., *Construction of improved Escherichia-Pseudomonas shuttle vectors derived from pUC18/19 and sequence of the region required for their replication in Pseudomonas aeruginosa.* Gene, 1994. **148**(1): p. 81-6.
3. Long, Y., et al., *Identification of novel genes that promote persister formation by repressing transcription and cell division in Pseudomonas aeruginosa.* J Antimicrob Chemother, 2019. **74**(9): p. 2575-2587.
4. Schweizer, H.P., *Allelic exchange in Pseudomonas aeruginosa using novel ColE1-type vectors and a family of cassettes containing a portable oriT and the counter-selectable Bacillus subtilis sacB marker.* Mol Microbiol, 1992. **6**(9): p. 1195-204.

Table S2. Primers used in this study.

Primer[a]	Sequence 5'-3'	Use
lpxT-UF	TATGACCATGATTACGAATTCGAAGCTGCTG CAGCAGCAG	pEX18Tc-*lpxT*
lpxT-UR	GGTCCTTGATTTCCCACACGCGGAACGGAGA GGCATTATCC	
lpxT-DF	GGATAATGCCTCTCCGTTCCGCGTGTGGGAA ATCAAGGACC	
lpxT-DR	ACGACGGCCAGTGCCAAGCTTCGATGACAG TCGCGAGACAG	
pmrB-UF	CGGGATCCAACTGCCTACCGGAGTCCCC	pEX18Tc-$pmrB_{L189Q}$
pmrB-UR	GCACTTCGCGTAGCGGCGCTTGGCCGCGGGC AACGCCGAA	
pmrB-DF	TTCGGCGTTGCCCGCGGCCAAGCGCCGCTAC GCGAAGTGC	
pmrB-DR	CCAAGCTTGCTAGCCTATCCCTTTCCCGGCC	
arnT-UF	CGGAATTCAGCTTCTACGGCGACGGCTACC	pEX18Tc-*arnT*
arnT-UR	GCAGGATCGCCAGGACCAGGTTTCATGGCT	
arnT-DF	CTGGGAGTGCGCCAGCCATGAAACCTGGTC	
arnT-DR	CCAAGCTTTGAATTGCACCCGGCCGTGTTC	
eptA-UF	AGCTCGGTACCCGGGGATCCATCTTCAGCGG	pEX18Tc-*eptA*
eptA-UR	GCCAGACGCTTCCTCGGAGTACAGTTTCGAC	
eptA-DF	GTGAAGATCCGTGCCCATGTCGAAACTGTAC	
eptA-DR	CGACGGCCAGTGCCAAGCTTCAAAGCGTCG	
P_{lpxT}-F	CGGAATTCGTAGTTGGACAGGCATTCCGC	P_{lpxT}-*lacZ*
P_{lpxT}-R	CGGGATCCAAAATCGTTTCCCCCTATCGGC	
lpxT-Flag-F	CGGAATTCGTAGTTGGACAGGCATTCCGC	E1553-*lpxT*-Flag
lpxT-Flag-R	CGACGGCCAGTGCCAAGCTTGATTACAAGG ACGACGATGACAAGGGACTGAGAAGCGCCG ATCT	
P_{lpxT}-*lpxT*-F	CCAAGCTTGTAGTTGGACAGGCATTCCGC	pUC18T-miniTN7T-*lpxT*
P_{lpxT}-*lpxT*-R	CGGGATCCGGACTGAGAAGCGCCGATCT	
lpxT-His-F	CGGGATCCTCGTGACAGTCGTACGCCGAC	pUCP20-*lpxT*-His
lpxT-His-R	CCAAGCTTTCAATGGTGATGGTGATGATGGA TGGCGGCCGAGTCGCGGACCAC	
$lpxT_{mut}$-His-F	GACCATGATTACGAATTCTCGTGACAGTCGT ACGCCGAC	pUCP20-$lpxT_{mut}$-His
$lpxT_{mut}$-His-R	ACGGCCAGTGCCAAGCTTTCAATGGTGATGG TGATGATGGATGGCGGCCGAGTCGCGGACC [illegible]	
$lpxT_{mut}$-F	GGTACCGGGCCCAAGCTTAGTAGTTGGACA GGCATTCCGC	pUC18T-miniTN7T-*l*

lpxT$_{\mathrm{mut}}$-R	GAGCTCACTAGTGGATCCGGACTGAGAAGCGCCGATCT	*pxT*$_{\mathrm{mut}}$
lpxT$_{\mathrm{G52A}}$-R	GTTTCGTTGAGGGCGAGGGATGCGTTGAGCTTGTCGAAGAGC	pUCP20-*lpxT*$_{\mathrm{G52A}}$-His&pUCP20-*lpxT*$_{\mathrm{G52A\ K137A}}$-His&pUC18T-miniTN7T-*lpxT*$_{\mathrm{G52A}}$&pUC18T-miniTN7T-*lpxT*$_{\mathrm{G52A\ K137A}}$
lpxT$_{\mathrm{G52A}}$-F	GCTCTTCGACAAGCTCAACGCATCCCTCGCCCTCAACGAAAC	
lpxT$_{\mathrm{K137A}}$-R	GTGGTCGCTGAGGTGGATGGCTGCGTCCATCAGCATCGACAGG	pUCP20-*lpxT*$_{\mathrm{K137A}}$-His&pUCP20-*lpxT*$_{\mathrm{G52A\ K137A}}$-His&pUC18T-miniTN7T-*lpxT*$_{\mathrm{K137A}}$&pUC18T-miniTN7T-*lpxT*$_{\mathrm{G52A\ K137A}}$
lpxT$_{\mathrm{K137A}}$-F	CCTGTCGATGCTGATGGACGCAGCCATCCACCTCAGCGACCAC	
lpxT$_{\mathrm{A181G}}$-R	GGAACTGCCCGGCGCGGCGTCCGAACAGCGACATGAAC	pUCP20-*lpxT*$_{\mathrm{A181G}}$-His&pUC18T-miniTN7T-*lpxT*$_{\mathrm{A181G}}$
lpxT$_{\mathrm{A181G}}$-F	GTTCATGTCGCTGTTCGGACGCCGCGCCGGGCAGTTCC	
arnT-Flag-F	CGGAATTCCTGCCGCTGGCCGAACTGGT	pMMB67EH-*arnT*-Flag
arnT-Flag-R	CCAAGCTTTCACTTGTCGTCATCGTCTTTGTAGTCGCGGCGCTCATGGGCGCAC	
lpxO1-Flag-F	CGGAATTCGCATACTCAGGCACTGAGAACAGGAG	pMMB67EH-*lpxO1*-Flag
lpxO1-Flag-R	CCAAGCTTTCACTTGTCGTCATCGTCTTTGTAGTCGATCCACAGGATGAAAGCCAGTACCAG	
lpxO2-Flag-F	CGGGATCCACCCGCAGGCACTCCTCC	pMMB67EH-*lpxO2*-Flag
lpxO2-Flag-R	CCAAGCTTTCACTTGTCGTCATCGTCTTTGTAGTCGCCGAAGATCCAACGATAGAGGATGAC	
eptA-Flag-F	CGGAATTCGGAAGTCAGTGAAGATCCGTGCCC	pMMB67EH-*eptA*-Flag
eptA-Flag-R	CCAAGCTTTCACTTGTCGTCATCGTCTTTGTAGTCGGAAGCCGGCGGCTCCTG	
qPCR primer		
q*lpxT*-F	GCGCAAGTTCGCCATCTGTC	RT-qPCR of *lpxT*
q*lpxT*-R	GCCAGAAGTCGAAGCCGTTC	
q*arnT*-F	GTGCTGCTGCGGATCAACAG	RT-qPCR of *arnT*
q*arnT*-R	GGATCGCCAGGACCAGGTTG	

a: F: forward; R, reverse; U, upstream of specific gene; D, downstream of specific gene; q, qPCR.
